# The prevalence and prescribing patterns of benzodiazepines and Z-drugs in older nursing home residents in different European countries and Israel: retrospective results from the EU SHELTER study

**DOI:** 10.1186/s12877-021-02213-x

**Published:** 2021-04-26

**Authors:** Anna Lukačišinová, Daniela Fialová, Nancye May Peel, Ruth Eleanor Hubbard, Jovana Brkic, Graziano Onder, Eva Topinková, Jacob Gindin, Tamar Shochat, Leonard Gray, Roberto Bernabei

**Affiliations:** 1 Department of Social and Clinical Pharmacy, Faculty of Pharmacy in Hradec Králové, Charles University, Akademika Heyrovského 1203, 500 05 Hradec Králové, Czech Republic; 2 Department of Geriatrics, 1st Faculty of Medicine, Charles University, Prague, Czech Republic; 3 Centre for Health Services Research, The University of Queensland, Brisbane, Australia; 4 Department of Cardiovascular, Endocrine-Metabolic Diseases and Aging, Istituto Superiore di Sanità, Rome, Italy; 5 The Center for Standards in Health and Disability, The University of Haifa, Haifa, Israel; 6 The Cheryl Spencer Department of Nursing, The University of Haifa, Haifa, Israel; 7 Centro Medicina dell’Invecchiamento, Dipartimento di Scienze Gerontologiche, Geriatriche e Fisiatriche, Universita Cattolica Sacro Cuore, Rome, Italy

**Keywords:** Benzodiazepines, Z-drugs, Nursing homes, Europe, Israel, Aged

## Abstract

**Background:**

Benzodiazepines (BZDs) and Z-drugs have high potential for developing frequent adverse drug events in older adults (e.g., psychomotor sedation, drug-related dementia, deliria, drug dependence, etc.). Knowledge of the prevalence and patterns of the use of BZDs/Z-drugs in vulnerable older patients is important in order to prevent and reduce the burden caused by their drug-related complications. Our study focused on international comparisons of the prevalence, country-specific prescribing patterns and risk factors of regular BZD/Z-drug use in nursing home (NH) residents.

**Methods:**

This cross-sectional study retrospectively analysed data of 4156 NH residents, prospectively assessed in the Services and Health in the Elderly in Long TERm care (SHELTER) project conducted from 2009 to 2014. Residents aged 65+ in 57 NHs in 7 European countries and Israel were assessed by the InterRAI Long-Term Care Facilities instrument. Descriptive statistics and multiple logistic regression models were used to describe the country-specific prevalence, patterns and risk factors of BZD/Z-drug use.

**Results:**

The mean age of the participants was 83.4 ± 9.4 years, 73% were female and 27.7% used BZDs/Z-drugs. The prevalence of BZD/Z-drug use differed significantly across countries, ranging from 44.1% in Israel to 14.5% in Germany. The most frequently prescribed were zopiclone (17.8%), lorazepam (17.1%) and oxazepam (16.3%). Lorazepam, oxazepam and diazepam were used in most of the countries. Brotizolam, temazepam and zolpidem showed highest prevalence in Israel (99.4% of all regular users of this medication in the sample), the Netherlands (72.6%) and France (50.0%), respectively. Residing in Israel was the most significant factor associated with the use of BZDs/Z-drugs or BZDs only (odds ratio [OR] 6.7; 95% confidence interval [CI] 4.8–9.2 and OR 9.7, 95%CI 6.5–14.5, respectively). The use of Z-drugs only was most significantly associated with residing in France (OR 21.0, 95%CI 9.0–48.9).

**Conclusions:**

Despite global recommendations and warnings, the preference for and extent of use of individual BZDs and Z-drugs in vulnerable NH residents differ significantly across countries. The strong association with country of residence compared to clinical and functional factors denotes that prescribing habits, social, cultural, behavioural, and regulatory factors still play an important role in the current diverse use of these medications.

**Supplementary Information:**

The online version contains supplementary material available at 10.1186/s12877-021-02213-x.

## Background

The nursing home (NH) environment is considered a specific setting because of the high prevalence of polymorbidity, polypharmacy, disability and geriatric frailty among NH residents, as well as because of a higher prevalence of geriatric syndromes and potentially inappropriate prescribing [1, 2]. Variation in population structure, criteria for admission to NHs and various prescription policies across different countries mostly result in significant differences in the number and nature of medications prescribed.

Common geriatric symptoms and syndromes (e.g., insomnia, depression, dementia, anxiety, behavioural and psychological symptoms of dementia, falls, etc.) [3] are associated with adverse drug outcomes, reduced quality of life [4] and increasing costs of care [5]. Among psychotropic medications, particularly the use of BZDs/Z-drugs is very frequent in older patients and this significantly contributes to a higher occurrence of drug-related geriatric symptoms and syndromes [3, 6].

BZDs are mostly prescribed for anxiety disorders, as adjuvant therapy for depression associated with anxiety, and in some clinical situations also as muscle relaxants [7]. They are also frequently prescribed for the treatment of behavioural and psychological symptoms of dementia despite the lack of appropriate evidence [8]. The common practice of prescribing of BZDs for the treatment of insomnia should have been substantially reduced in geriatric patients already a long time ago [9].

On the other hand, the usual indication for Z-drugs (zolpidem, zopiclone, zaleplon and eszopiclone) is the first-line pharmacological strategy for the treatment of insomnia [10]. However, long-term use of these medications should be avoided in older adults and substituted, if necessary, by alternative hypnotics (e.g., mirtazapine, trazodone, etc.) and always supported by non-pharmacological approaches (e.g., cognitive behavioural therapy, relaxation therapy, sleep hygiene, psycho-social support, etc.) [9, 11]. Due to differences in the prevalence of psychiatric disorders and in the appropriateness of prescribing of various drugs and drug classes, the proportion of BZD prescription in NHs in different studies differs widely coming up to 54% [3, 12]. The prevalence of Z-drugs in the older population was mostly reported up to 23% [13, 14].

There is great concern about the use of BZDs and Z-drugs in older patients, particularly in vulnerable older adults in the NH environment. Evidence on the effectiveness of these medications, especially for the long-term treatment of insomnia, is questionable [15]. On the other hand, their potential for adverse drug events including various geriatric syndromes (e.g., falls and fractures, cognitive impairment, functional decline, psychomotor sedation, orthostasis and delirium) has been well described [13, 16–23]. Additionally, there is strong evidence of a high risk of developing drug dependence [20]. Also, frequent co-prescribing of BZDs and opioids should be of particular concern given the extent to which this combination contributes to excessive sedative effect, pharmaceutical overdosing and deaths [21].

Older adults in the NH setting represent a population with a complex burden of polymorbidity, polypharmacy, geriatric frailty and frequent psychological and social problems. They can be considered a group that is highly vulnerable to adverse drug events associated with BZDs/Z-drugs [22]. For these reasons, monitoring trends in the use of BZDs/Z-drugs in older adults, is important, as is monitoring the rationality of their indications, dosing, use in combined drug regimens and length of therapy.

Previous, mostly country-specific studies reported a frequent use of BZDs/Z-drugs in the NH setting. Since these studies used different tools and instruments for data collection and various techniques of data evaluation, making direct comparisons of these rather diverse results is not possible. Moreover, no international comparisons of the patterns of BZD/Z-drug use in NHs at the European level of this extent have been published yet.

Therefore, the objective of our study was to determine and compare the prevalence and prescribing patterns of regularly used BZDs/Z-drugs in NH residents in 7 European countries and Israel using standardised data obtained from the EU SHELTER project (Services and Health in the Elderly in Long TERm care project, 2009–2014), collected under the 7th Framework Programme of the European Commission (FP7-HEALTH). We focused particularly on describing and explaining specific prescribing patterns of the regular use of BZDs/Z-drugs in NH residents in participating country samples and on determining factors influencing the regular use of these medications.

## Methods

### Study design

Data were collected as a part of the EU SHELTER project that prospectively assessed 4156 NH residents aged 65 years and older in 7 European Union (EU) countries (the Czech Republic, England, Finland, France, Germany, Italy, and the Netherlands) and 1 non-EU country (Israel). The SHELTER study originally aimed to validate the interRAI instrument for Long-Term Care Facilities (interRAI LTCF) as a tool to assess care needs and the provision of care to NH residents in Europe. The baseline assessment of NH residents was conducted from 2009 to 2011, followed by two reassessments. The data were further cleaned and analysed for different goals mostly after 2014. NH partners willing to participate in the study were identified in each country. The sample was not randomly selected, and it was not intended to be representative of all NH residents in each country. Overall, 57 NH facilities participated in the study (the Czech Republic: 10, England: 9, Finland: 4, France: 4, Germany: 9, Israel: 7, Italy: 10, the Netherlands: 4) accounting for 450–500 residents per country. Older adults who had been residing long-term in the participating NH facilities at the beginning of the study and those who had been admitted for a long-term stay in the 3-month enrolment period following the initiation of the study were included in the study. No further exclusion criteria except those arising from selected inclusion criteria were applied. Participants enrolled in the study were assessed using the interRAI LTCF instrument and re-assessed at 6- and 12-month periods if they were still in the facility. If they were no longer in the facility, the reason (death, hospitalisation, discharge to home or to other institutions) and date of death or discharge were recorded. Our analysis used data collected at the baseline period of the study because of the expected and confirmed high drop-out rates of study participants in reassessment periods. A complete description of the project methodology was published by Onder et al., 2012 [23].

### Ethics

Ethical approval was obtained from the ethical committees of all participating countries in accordance with local regulations, and only those patients who signed informed consent to participate in the study were included in the SHELTER project. Participating subjects were free to decline participation any time during the course of the study and data were collected and stored under specific codes with an assurance of anonymity and data confidentiality.

### Data collection

The interRAI LTCF assessment instrument was used for data collection. It is a setting-specific tool developed by the interRAI corporation (www.interrai.org) that has been standardised and validated in previously published studies over the past decade [24]. It comprises over 350 data elements including patient-related characteristics (socio-demographic, clinical status, physical and cognitive status information, medical diagnoses, symptoms, signs, patient medication information) and care-related characteristics (provision of specific services, informal care, time burden of care, etc.). There are number of clinical and functional scales embedded within the interRAI LTCF instrument that have been previously tested, standardised and validated. Of those, to describe the functional status of NH residents we used the Activities of Daily Living Hierarchy scale (ADLH) [25], while the cognitive status of NH residents was measured using the Cognitive Performance Scale (CPS) [26]. Depression and its severity were documented by the Depression Rating Scale (DRS) [27]. The Pain Scale [28] was applied to describe the presence and intensity of pain, and the communication abilities of the patients were measured by the Communication Scale [29]. The level of consciousness was evaluated using the Clinical Assessment Protocol (CAP) for delirium [30]. For a detailed description of geriatric scales, their ranges and clinically significant cut-off points for diagnostic issues and analyses, please see the footnotes to Tables 1 and 2.

**Table 1:** Description of main patient characteristics and comparisons between of regular BZD/Z-drug users and non-users

	Total sample *N* = 4023 (%)	All regular BZD/Z-drug users *N* = 1113 (%)	BZD/Z-drug non-users *N* = 2910 (%)	***p***-value
**Age, years** mean ± SD	83.5 ± 9.4	83.2 ± 9.4	83.7 ± 9.3	0.10
**Age categories N (%)**				
≤ 65 years	177 (4.0)	51 (5.0)	126 (4.7)	0.267
66–74 years	354 (9.6)	108 (10.7)	246 (9.1)	
75–84 years	1172 (31.7)	330 (32.7)	842 (31.3)	
≥ 85 years	1197 (54.0)	521 (51.6)	1476 (54.9)	
**Gender** (female)	2945 (73.2)	822 (73.9)	2123 (73.0)	0.53
**Number of medications** mean ± SD	7 ± 3.6	8.3 ± 3.3	6.5 ± 3.6	**< 0.001**
**Number of medications categories**				
≤ 4 medications	1044 (26.0)	144 (12.9)	900 (30.9)	**< 0.001**
5–9 medications	2000 (49.7)	581 (52.2)	1419 (48.8)	
≥ 10 medications	979 (24.3)	388 (34.9)	591 (20.3)	
**CPS** ^**a**^				
Mean ± SD	2.9 ± 1.9	2.7 ± 1.9	3.0 ± 1.9	**0.002**
Median (IQR)	3.0 (1.0–5.0)	3.0 (1.0–5.0)	3.0 (1.0–5.0)	
**CPS categories** N (%)				
0–1 Cognition intact	1116 (28.3)	367 (33.5)	796 (26.3)	**< 0.001**
2–6 Cognition impaired	2831 (71.7)	730 (66.5)	2101 (73.7)	
**ADL Hierarchy scale** ^**b**^				
Mean ± SD	15.2 ± 9.5	14.2 ± 9.7	15.5 ± 9.5	**< 0.001**
Median (IQR)	16.0 (7.0–24.0)	15.0 (5.0–23.0)	17.0 (7.0–24.0)	
**ADL Hierarchy scale Categories** N (%)				
0–1 Independent	744 (18.5)	254 (22.8)	490 (16.9)	**< 0.001**
2–4 Assistance required	1661 (41.4)	445 (40.0)	1216 (41.9)	
5–6 Dependent	1607 (40.0)	413 (37.1)	1194 (41.2)	
**PAIN scale** ^**c**^				
Mean ± SD	0.6 ± 0.8	0.7 ± 0.9	0.5 ± 0.8	**< 0.001**
Median (IQR)	0.0 (0.0–1.0)	0.0 (0.0–1.0)	0.0 (0.0–1.0)	
**PAIN scale categories** N (%)				
0 No pain	2280 (61.2)	550 (55.2)	1730 (63.4)	**< 0.001**
1 Mild pain	922 (24.8)	281 (28.2)	641 (23.5)	
2 Moderate pain	395 (9.8)	110 (11.0)	285 (10.5)	
3 Severe pain	109 (2.7)	49 (4.9)	60 (2.2)	
4 Excruciating pain	17 (0.4)	6 (0.6)	11 (0.4)	
**CAP Delirium** ^d^				
Mean ± SD	0.3 ± 0.8	0.3 ± 0.9	0.3 ± 0.8	0.18
Median (IQR)	0.0 (0.0–0.0)	0.0 (0.0–0.0)	0.0 (0.0–0.0)	
**Depression scale** ^e^				
Mean ± SD	2.1 ± 2.7	2.6 ± 2.9	1.9 ± 2.6	**< 0.001**
Median (IQR)	1.0 (0.0–3.0)	2.0 (0.0–4.0)	1.0 (0.0–3.0)	
**Depression scale Categories** N (%)				
0 No clinical problems	1645 (41.5)	379 (34.5)	1266 (44.3)	**< 0.001**
1–2 Changes in mood, but not clinically relevant depression	1048 (26.5)	284 (25.8)	764 (26.7)	
≥ 3 Clinically relevant depression	1268 (32.0)	437 (39.7)	831 (29.0)	
**Communication scale** ^f^				
Mean ± SD	3.0 ± 2.9	2.6 ± 2.8	3.1 ± 2.9	**0.05**
Median (IQR)	2.0 (0.0–6.0)	2.0 (0.0–5.0)	2.0 (0.0–6.0)	
**Communication scale Categories** N (%)				
0 Intact	1736 (42.3)	525 (47.6)	1146 (40.0)	**< 0.001**
1–4 Mild to moderate	1092 (26.6)	279 (25.3)	776 (27.1)	
5–8 Moderate-severe to very severe	1273 (30.6)	299 (27.1)	943 (32.9)	

Results in bold indicate statistically significant results

^a^ CPS – Cognitive Performance Scale [26] was used to access cognitive status. It includes five items: cognitive skills for daily decision making, short-term memory problems, procedural memory problems, making self-understood, and eating ability. Scores of CPS items range from 0 (intact) to 6 (very severe cognitive impairment), and any score ≥ 2 indicates clinically significant cognitive impairment (from mild to very severe stages)

^b^ ADL Hierarchy scale – Activities of Daily Living Hierarchy scale [25] comprises 7 items: personal hygiene, dressing upper body, dressing lower body, locomotion, toilet use, bed mobility, eating. Each item is scored from 0 = requires supervision to 4 = total dependence. The scale ranges from 0 to 28, with higher scores reflecting greater level of dependency and difficulties in performing activities

^c^ Pain scale [28] - summarizes the reported presence and intensity of pain. The scores range from 0 = no pain to 4 = daily excruciating pain

^d^ CAP Delirium [30] – this scale comprises 4 items: easily distracted, disorganized speech, mental function varies over day, change in decision making. The scale ranges from 0 to 4, with higher values indication increase likelihood of delirium

^e^ Depression scale [27] – is based on the self-reported mood items and indicates the presence of depressed mood and anxiety. It consists of 3 self-reported mood items, while each question can be scored from 0 to 2 with the maximum overall score of 6. The score of this scale range from 0 = no symptoms of depression to 6 = all symptoms present in last 3 days/24 h: high likelihood of depression

^f^ Communication scale [29] – consists of two items: making self-understood (expression) and ability to understand others (comprehension), while not taking directly into consideration hearing and visual impairment. It is primarily focused on dysphasia and similar syndromes. The scores range from 0 = intact to 8 = very severe impairment

**Table 2:** Factors influencing regular use of BZDs/Z-drugs, regular use of BZDs only, and regular use of Z-drugs only in the studied sample – results from the multiple logistic regression models

**Factors influencing regular use of drug groups**	**BZDs/Z-drugs**			**BZDs only**			**Z-drugs only**	
	**Adjusted** **OR (95% CI)** ^**g**^	***p*** **value**		**Adjusted** **OR (95% CI)** ^**g**^	***p*** **value**		**Adjusted** **OR (95% CI)** ^**g**^	***p*** **value**
**Age**	0.993 (0.985–1.002)	0.139		**0.990** **(0.980–0.999)**	**0.029**		1.001 (0.986–1.017)	0.858
**Gender**								
Male – reference	1.000			1.000			1.000	
Female	1.063 (0.885–1.277)	0.513		1.061 (0.870–1.295)	0.558		1.073 (0.783–1.471)	0.661
**Countries ordered by increasing prevalence of regular use of BZDs/Z-drugs**			**Countries ordered by increasing prevalence of regular use of BZDs only**			**Countries ordered by increasing prevalence of regular use of Z-drug only**		
Germany – ref.	1.000		England – ref.	1.000		Italy – ref.	1.000	
England	**1.532** **(1.070–2.193)**	**0.020**	Germany	0.971 (0.560–1.503)	0.731	Finland	1.311 (0.487–3.527)	0.592
The Czech Republic	**1.509** **(1.064–2.140)**	**0.021**	The Czech Republic	**1.857** **(1.192–2.894)**	**0.006**	The Netherlands	1.093 (0.415–2.880)	0.857
Finland	**1.888** **(1.324–2.691)**	**< 0.001**	Finland	**3.298** **(2.146–5.068)**	**< 0.001**	The Czech Republic	**3.662** **(1.570–8.542)**	**0.003**
Italy	**2.631** **(1.857–3.727)**	**< 0.001**	France	**3.655** **(2.226–6.002)**	**< 0.001**	Germany	**3.809** **(1.655–8.769)**	**0.002**
The Netherlands	**2.424** **(1.738–3.381)**	**< 0.001**	Italy	**4.368** **(2.864–6.622)**	**< 0.001**	Israel	**5.620** **(2.478–12.744)**	**< 0.001**
France	**5.250** **(3.473–7.936)**	**< 0.001**	The Netherlands	**4.008** **(2.700–6.164)**	**< 0.001**	England	**7.727** **(3.428–17.420)**	**< 0.001**
Israel	**6.660** **(4.823–9.198)**	**< 0.001**	Israel	**9.715** **(6.501–14.517)**	**< 0.001**	France	**20.953** **(8.970–48.940)**	**< 0.001**
**CPS**^**a**^	0.974 (0.908–1.044)	0.459		0.953 (0.883–1.030)	0.224		0.998 (0.892–1.115)	0.965
**ADLH**^**b**^	0.992 (0.981–1.003)	0.151		0.991 (0.979–1.005)	0.131		0.993 (0.976–1.012)	0.479
**Pain scale**^**c**^	**1.113** **(1.004–1.234)**	**0.041**		**1.119** **(1.000–1.252)**	**0.050**		1.095 (0.926–1.294)	0.290
**CAP Delirium**^**d**^	0.992 (0.897–1.097)	0.876		1.012 (0.910–1.125)	0.830		0.884 (0.715–1.092)	0.253
**Depression scale**^**e**^	**1.052** **(1.020–1.085)**	**0.001**		**1.064** **(1.030–1.100)**	**< 0.001**		1.012 (0.960–1.067)	0.659
**Communication scale**^**f**^	0.941 (0.898–0.986)	**0.010**		0.955 (0.907–1.005)	0.077		0.930 (0.861–1.006)	0.069
**Anxiety**^**h**^								
Not present – reference	1.000			1.000			1.000	
Diagnosis present	1.171 (0.608–2.252)	0.637		1.274 (0.639–2.540)	0.491		1.162 (0.407–3.323)	0.779
Diagnosis present, treated	**1.887** **(1.382–2.578)**	**< 0.001**		**2.320** **(1.688–3.187)**	**< 0.001**		0.584 (0.316–1.078)	0.085
Diagnosis present, monitored	0.820 (0.525–1.280)	0.382		0.792 (0.492–1.275)	0.337		0.957 (0.423–2.167)	0.917
**Difficulty falling asleep**								
Not present – reference	1.000			1.000			1.000	
Diagnosis present in medical record, problem not exhibited	**2.687** **(2.098–3.443)**	**< 0.001**		**2.392** **(1.837–3.115)**	**< 0.001**		**2.767** **(1.859–4.119)**	**< 0.001**
Exhibited 1 of 3 days	**1.953** **(1.315–2.901)**	**< 0.001**		1.501 (0.977–2.306)	0.063		**3.052** **(1.647–5.654)**	**< 0.001**
Exhibited 2 of 3 days	**1.777** **(1.118–2.824)**	**0.015**		**1.659** **(1.007–2.731)**	**0.047**		1.395 (0.578–3.370)	0.459
Exhibited daily of 3 days	**3.274** **(2.481–4.320)**	**< 0.001**		**2.426** **(1.797–3.275)**	**< 0.001**		**3.526** **(2.373–5.238)**	**< 0.001**

Results in bold indicate statistically significant results

^a^ CPS– Cognitive Performance Scale [26] was used to access cognitive status. It includes five items: cognitive skills for daily decision making, short-term memory problems, procedural memory problems, making self-understood, and eating ability. Scores of CPS items range from 0 (intact) to 6 (very severe cognitive impairment), and any score ≥ 2 indicates clinically significant cognitive impairment (from mild to very severe stages)

^b^ ADLH scale –Activities of Daily Living Hierarchy scale [25] comprises 7 items: personal hygiene, dressing upper body, dressing lower body, locomotion, toilet use, bed mobility, eating. Each item is scored from 1 = requires supervision to 4 = total dependence. The scale ranges from 0 to 28, with higher scores reflecting greater level of dependency and difficulties in performing activities

^c^ Pain scale [28] - summarizes the reported presence and intensity of pain. It comprises two items: pain symptoms-frequency and pain symptoms-intensity of highest level of pain present. The scores range from 0 = no pain to 4 = daily excruciating pain

^d^ CAP Delirium [30] - this scale comprises 4 items: easily distracted, disorganized speech, mental function varies over day, change in decision making. The scale ranges from 0 to 4, with higher values indication increase likelihood of delirium

^e^ Depression scale [27] - is based on the self-reported mood items and indicates the presence of depressed mood and anxiety. It consists of 3 self-reported mood items, while each question can be scored from 0 to 2 with the maximum overall score of 6. The score of this scale range from 0 = no symptoms of depression to 6 = all symptoms present in last 3 days/24 h: high likelihood of depression

^f^ Communication scale [29] – consists of two items: making self-understood (expression) and ability to understand others (comprehension), while not taking directly into consideration hearing and visual impairment. It is primarily focused on dysphasia and similar syndromes. The scores range from 0 = intact to 8 = very severe impairment

^g^ Adjusted for all factors in univariate logistic regression: age, gender, functional and cognitive status, anxiety, insomnia, depression, delirium, pain, and communication problems

^h^ “Diagnosis present” – recorded when diagnosis confirmed as diagnosed clinical condition in medical charts; “Diagnosis present, treated” – resident’s diagnosis is being treated by active treatment (incl. Drug therapy, therapeutic rehabilitation services, other medical or skilled nursing interventions); “Diagnosis present, monitored” – resident’s diagnosis is being only monitored (e.g., by laboratory tests, vital signs, etc.) but no active treatment is provided

Drug information collected included all medications the patients had been taking in the 3 days prior to the assessment. Clinical and medication information were derived from multiple sources, medical charts, medication administration records and also using clarifications from healthcare staff (physicians and nurses) caring for the patients. The drug name (non-proprietary, proprietary), Anatomical Therapeutic and Chemical (ATC) code based on the WHO Collaborating Centre for Drug Statistics Methodology [31], formulation, dosage, frequency and route of administration were recorded in an electronic dataset. A complete and detailed description of data collection has been published elsewhere [23].

### Outcome measures

The primary outcomes of this study were to determine: (1) the prevalence of regular BZD/Z-drug use in older NH residents across samples of participating countries, (2) the prevalence portfolio of different BZDs/Z-drugs prescribed for regular use in analysed samples; and (3) to identify factors influencing the prescription of the regular use of BZDs/Z-drugs in older NH residents.

### Statistical analyses

To describe cross-sectional differences in the prevalence of BZD and Z-drug use and to capture all possible existing BZDs and Z-drugs prescribed, all existing ATC codes were analysed (see Additional Table 1). Only those medications administered regularly in different drug schemes (over the past 3 days) were analysed and medications used only occasionally (per re nata – PRN) were not included in the group of regular BZD/Z-drug users (in total, 327 patients, representing 8.1% of the total sample, for details see Additional Table 2). Of the 4156 older NH residents in the study database, 133 subjects were excluded due to missing data (missing medication records). A missing medication record was specified as one having missing medication records describing the characteristics of individual drugs prescribed, even if the total number of medications was recorded as a “non zero” value. Frequency distributions were used to describe the main characteristics of the studied population and the prevalence of BZD/Z-drug use. The relationship between the use of BZDs/Z-drugs and major patient-related characteristics was analysed using a univariate regression model. For continuous variables, the parametric (t-Test) or non-parametric (Mann-Whitney U Test) comparisons of means were used, depending on data distribution. For categorical data, Pearson’s Chi-Square Test was performed. Finally, multiple logistic regression models were used to identify predictive factors of the regular use of BZDs/Z-drugs, and also separately predictive factors of the regular use of only BZDs and only Z-drugs. All multiple regression models were adjusted for factors that were statistically significantly associated with BZD/Z-drug use confirmed in univariate logistic regression models, namely age, gender, functional and cognitive status, anxiety, insomnia, depression, delirium, pain, and communication problems. The level of statistical significance was set at *p* < 0.05 and all proportions were calculated as percentages of patients with available data. Analyses were performed using SPSS_ IBM Version 20 (SPSS, Inc., Chicago, IL, USA).

## Results

### Characteristics of BZD/Z-drug users and non-users

The mean age of the NH residents in the total population of 4023 patients (excluding those patients having missing medication data) was 83.5 ± 9.4 years and the majority of patients (73.2%) were women. The majority of patients were 85 years and older (54.0%). There was no significant difference between BZD/Z-drug users within age group categories. The mean number of medications used was 7.0 ± 3.6. Significantly more patients in the group of BZD/Z-drug users had ≥10 medications compared to non-users (34.9% vs. 20.3%, *p* < 0.001). A comparison of the basic characteristics between groups of users and non-users of BZDs/Z-drugs is shown in Table 1. In general, users of BZDs/Z-drugs suffered from pain of higher intensity and frequency and suffered from higher stages of clinically relevant depression. On the other hand, BZD/Z-drug users were less cognitively and functionally impaired and had fewer communication problems compared to non-users.

### The prevalence and patterns of BZD/Z-drug use

In the overall sample, 27.7% of patients were regularly prescribed at least 1 BZD/Z-drug. There were 1247 BZDs/Z-drugs regularly used in the sample. One hundred thirteen users of multiple BZDs/Z-drugs (users of different combinations of BZDs/Z-drugs) represented 10.2% of all regular BZD/Z-drug users. The three most frequently regularly prescribed BZDs were lorazepam, oxazepam and brotizolam (17.1, 16.3, 13.8%, respectively), while Z-drugs were represented mainly by zopiclone and zolpidem (17.8 and 11.7%, respectively). For details on the frequencies of BZD/Z-drug use and their combinations see Additional Table 2.

**Fig. 1:**
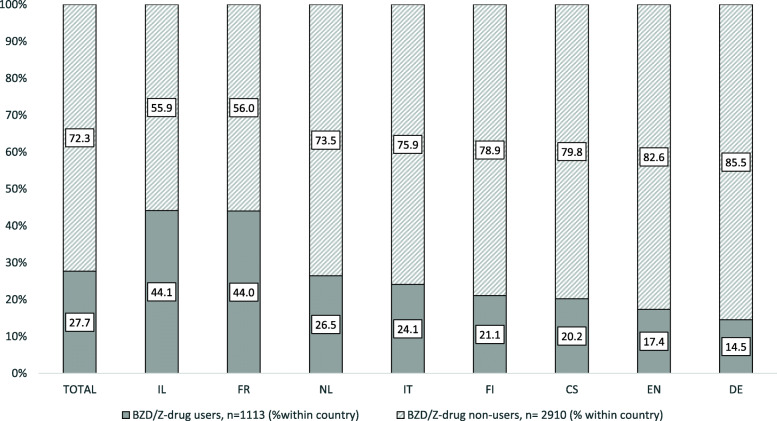
Prevalence (%) of BZD/Z-drug users across countries. IL-Israel, FR-France, NL-The Netherlands, IT-Italy, FI-Finland, CS-The Czech Republic, EN-England, GE-Germany

**Fig. 2:**
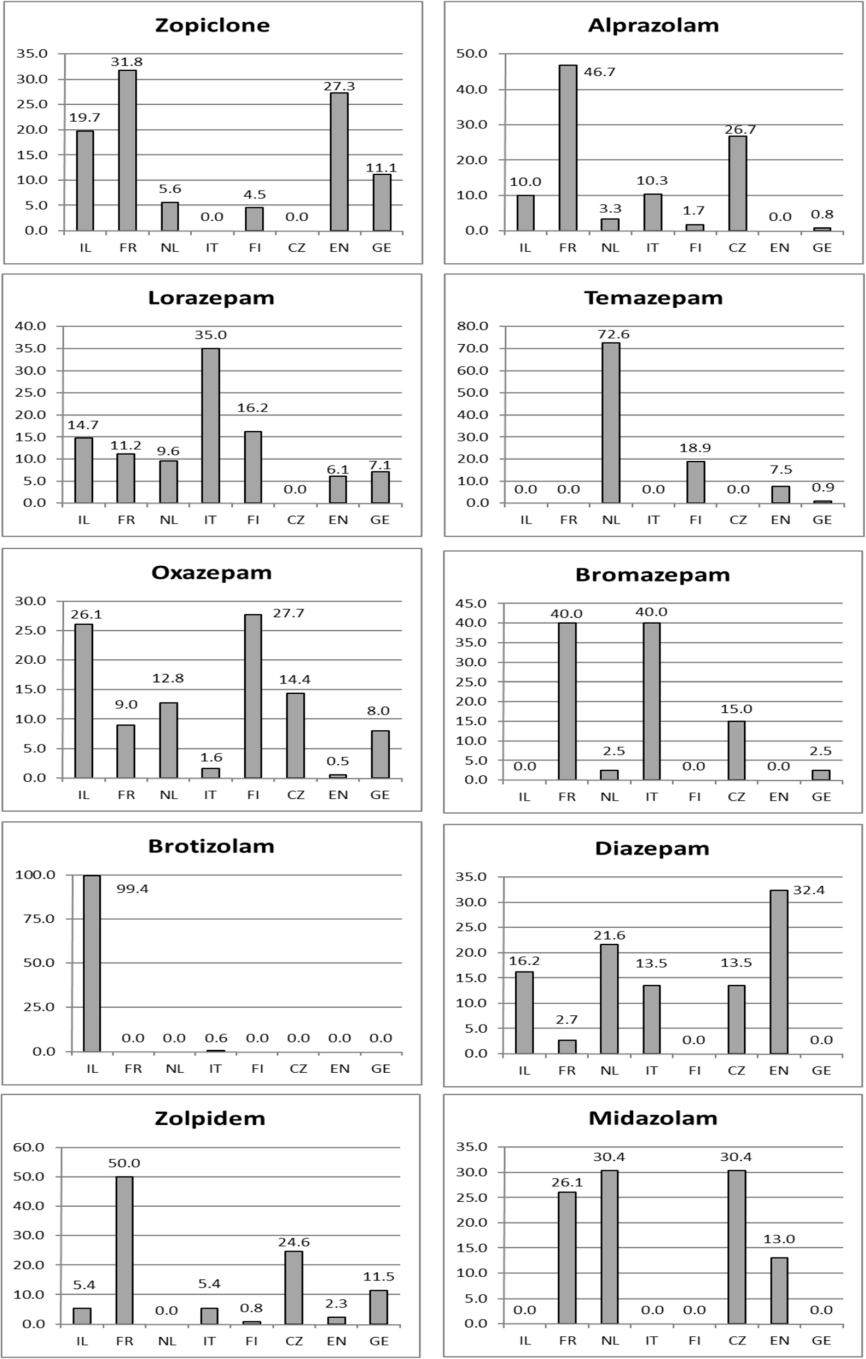
Distribution (%) of 10 most frequent BZDs and Z-drugs across countries. IL-Israel, FR-France, NL-The Netherlands, IT-Italy, FI-Finland, CS-The Czech Republic, EN-England, GE-Germany

**Fig. 3:**
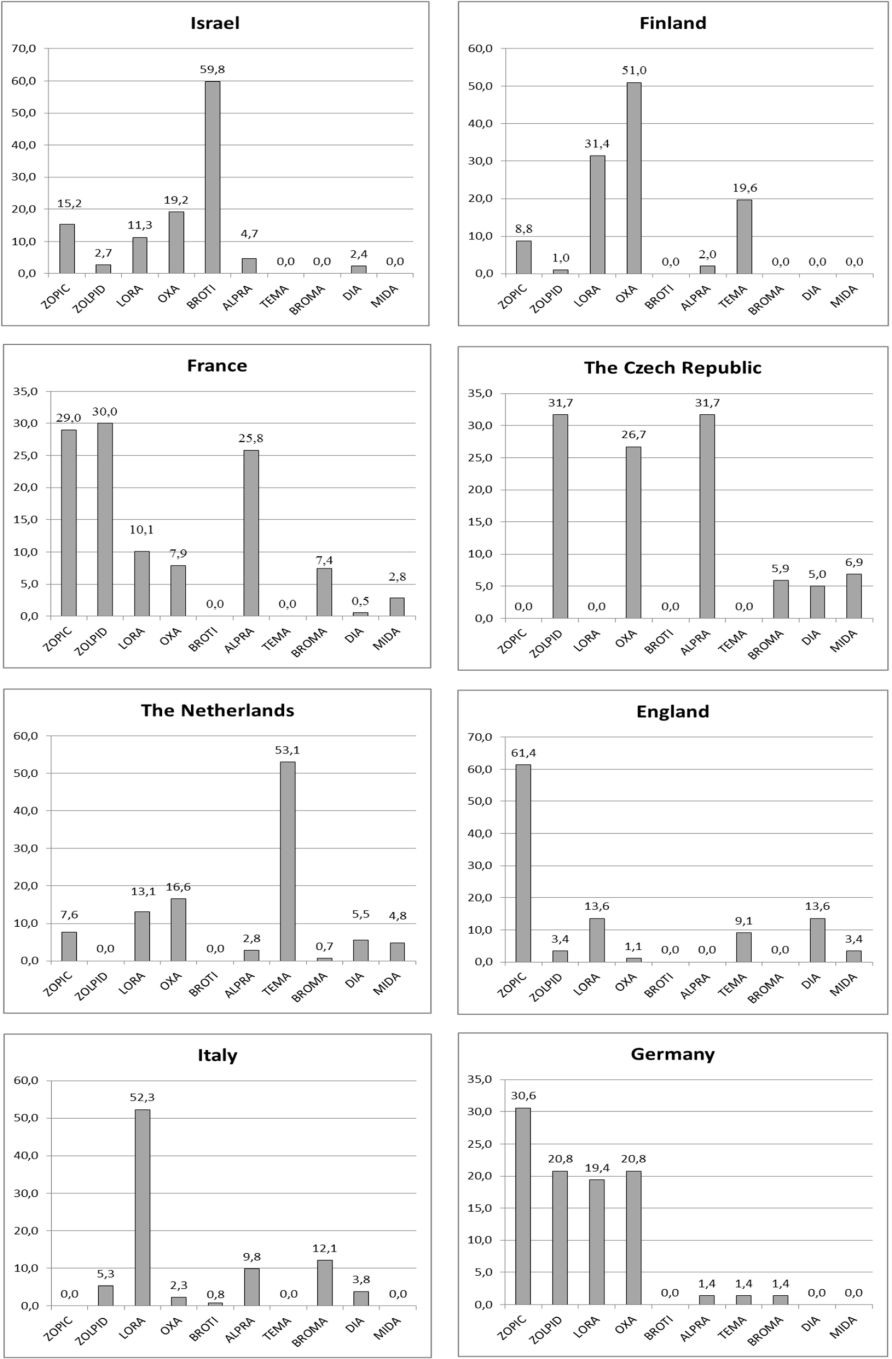
Distribution (%) of BZDs and Z-drugs within users in particular country. ZOPIC-zopiclone, ZOLPID-zolpidem, LORA-lorazepam, OXA-oxazepam, BROTI-brotizolam, ALPRA-alprazolam, TEMA-temazepam, BROMA-bromazepam, DIA-diazepam, MIDA-midazolam. The total percentage count can exceed 100% of users due to multiple users within and across different BZD/Z-drug users in each country

Figure 1 shows the prevalence of BZDs/Z-drugs in individual countries, with Israel having the highest (44.1%) and Germany the lowest (14.5%) prevalence of all regular BZD/Z-drug users. The differences in the prescribing patterns of the 10 most frequently used BZDs/Z-drugs are described in Fig. 2. Oxazepam was prescribed in all participating countries and alprazolam, lorazepam, diazepam, zopiclone, and zolpidem were prescribed in most of the participating countries (6–7 countries). The rest of identified BZDs were confirmed to be prescribed in fewer than 5 participating countries. Brotizolam, temazepam and zolpidem showed predominant use (≥ 50% of all regular users of this medication in the sample) in Israel, the Netherlands and France, respectively. Figure 3 demonstrates the prescribing patterns in all participating countries. In Israel, Finland, the Netherlands, England and Italy, one rather dominant BZD/Z-drug was prescribed (exceeding the prevalence of 50% of all users in the country). On the other hand, in France, the Czech Republic, and Germany a broader spectrum of BZDs/Z-drugs was used.

### Factors associated with the use of BZDs and Z-drugs

According to the unadjusted univariate logistic regression model (Additional Table 3), every one-point increase on the CPS scale, ADLH scale, and Communication Scale (denoting poorer performance) was associated with a lower probability of using BZDs/Z-drugs. However, these findings were not statistically significant after adjusting univariate results for other factors in the multiple logistic regression models. According to the results of multiple regression models (Table 2), every one-point increase on the Depression Scale (denoting poorer performance) was associated with higher probability of BZD/Z-drug use as well as with use of only BZDs. Patients with presence and treatment of anxiety were almost two times more likely to be prescribed BZDs/Z-drugs (OR 1.887, 95%CI 1.382–2.578, *p* < 0.001) and more than two times more likely to be prescribed only BZDs (OR 2.320, 95%CI 1.688–3.187, *p* < 0.001). Patients with difficulties falling asleep (exhibited daily of 3 days) had a significantly higher probability of using BZDs/Z-drugs, only BZDs as well as only Z-drugs. More importantly also patients with present diagnoses of insomnia in the medical record but with no problems exhibited in last 3 days were almost 3 times more likely to be prescribed regularly either of the analysed medication groups (OR for regular use of BZDs/Z-drugs was 2.687, 95%CI 2.098–3.443, *p* < 0.001, OR for regular use of BZDs only 2.392, 95%CI 1.837–3.115, *p* < 0.001, and for regular use of Z-drugs only 2.767, 95%CI 1.859–4.119, *p* < 0.001) (Table 2).

In all adjusted multiple logistic regression models, countries were ordered and tested according to increasing prevalence of regular use of a relevant medication group (Table 2). All three models showed a similar pattern in association of country of residence with the regular use of BZDs/Z-drugs, BZDs only and Z-drugs only. NH patients residing in countries with the higher prevalence of regular use of particular medication group had a higher probability to be prescribed a drug from this group. In the model of regular BZD/Z-drug users and in the model of only regular BZD users the highest OR were found for Israel (OR 6.660, 95%CI 4.823–9.198, *p* < 0.001 and OR 9.715, 95%CI 6.501–14.517, *p* < 0.001, respectively). In the model of regular Z-drug users only, the highest probability of being regularly prescribed Z-drugs was found in England and France (OR 7.727, 95%CI 3.428–17.420, *p* < 0.001 and OR 20.953, 95%CI 8.970–48.940, *p* < 0.001, respectively).

## Discussion

Our study is a larger retrospective cross-sectional study that analysed data prospectively collected from over 4000 NH residents in 7 European countries and Israel who participated in the SHELTER project. To the best of our knowledge, this study presents the broadest comparisons of prescribing patterns of the use of BZDs and Z-drugs in NH settings in Europe and Israel. Prior to now, no such extensive study using a comprehensive geriatric assessment methodology has been published in respect of vulnerable older adults in NH settings in European countries. In our study, the same methodological approach in all participating country samples was used. NH residents were assessed prospectively using standardised and validated geriatric scales embedded within the InterRAI-LTCF instrument. This design enabled us to uniquely incorporate a comprehensive geriatric assessment method into pharmacoepidemiologic evaluations of BZD/Z-drug use. As BZDs and Z-drugs are often found among those medications inappropriately over-prescribed in older patients, our results document important current evidence on the extent and patterns of use of these medications in the vulnerable geriatric population in European and Israeli NHs and confirm significant differences across the studied samples.

The overall prevalence of BZD/Z-drug use in the NH setting was 27.7%, which correlates with findings of other studies from various European countries [3, 12]. Mean age of patients in our sample corresponds to the recent study of European NH facilities (6 different European countries) reporting mean age of patients to be 83.9 ± 7.2 [32]. Also, the prevalence of women (72.3%) in our study is similar to findings of other multinational studies in NH settings (67%) [32].

In contrary to other studies [33], our analyses showed no statistically significant association between the use of BZDs/Z-drugs and age and gender. Some of the more recent findings of other studies have indicated advanced age (≥ 85 years) to be a protective factor for prescribing excessive polypharmacy [34], as well as a protective factor of prescribing potentially inappropriate medications (PIMs) in the aged [35]. While the evidence points to growing awareness of prescribing PIMs in very old patients, we only partially confirmed such findings. Even though the mean age of the older population in our sample was 83.5 ± 9.4 years and we documented such a protective effect in a univariate logistic regression model, this result was not statistically significant in multiple logistic regression models for BZDs/Z-drugs and only Z-drugs. On the other hand, in the multiple logistic regression model, very old age remained a protective factor for the use of BZDs (OR = 0.99, 95% CI 0.980–0.999; *p* = 0.029) when including only BZD users, even though the significance might be considered borderline. This finding might suggest recognition of a potential inappropriateness and adverse drug effects of BZDs in the advanced age group of seniors leading to deprescribing strategies in the regular use of BZDs in this age group.

Some studies of the older population have reported gender to have no statistically significant association with polypharmacy [34], nor with the prescription of PIMs [35]. However, opposite findings exist that show a predominant use of polypharmacy and PIMs in older females, thus the published evidence in this respect is conflicting [33]. In our study, there was no statistically significant association between the use of BZDs/Z-drugs and the female gender in any of our tested multiple logistic regression models.

The results of the univariate logistic regression also showed a negative association between cognition, functional, and communication performance and the use of BZDs/Z-drugs (Additional Table 3). These results did not remain statistically significant after adjusting for other potential factors in multiple logistic regression models. However, it is important to emphasise that the trends seen in the results of these factors remained similarly nonsignificant across all models (Table 2).

In terms of the association between the use of BZDs/Z-drugs and cognitive and functional impairment in older adults in other studies, the published research has yielded mixed results [36, 37] and some studies have showed that BZD use is significantly associated mainly with impairment in executive memory functioning [36, 38]. Here, the previously described protective effect of advanced age might also play a role, as advanced age is usually accompanied with cognitive, functional and communication performance decline [39].

In our study, the regular use of BZDs/Z-drugs was positively associated with the presence of diagnoses of depression and anxiety, as well as with the severity of depression and pain. As confirmed by all multiple logistic regression models, BZDs and Z-drugs were used also on regular daily basis in patients who had recorded a diagnosis of insomnia but had not exhibited any actual sleeping problems in the past 3 days. This as a warning finding and it should rise alert to regular long-term use of hypnosedatives in patients with not actually exhibited sleeping problems. As propensity to cause adverse drug effects [13, 16–21] and addictive potential [20] of these medication groups is well described, it should be of particular importance to drive caution to their regular use in frail older patients.

BZDs/Z-drugs are considered medications that are potentially inappropriate for the older population [1, 2], and their high potential to cause drug dependency when used regularly has been repeatedly confirmed [20]. Moreover, residents in NHs are often prescribed long-term medications without any sufficient feedback control, which is essential for early dis/continuation of therapy. Therefore, our analyses focused on regular use of BZDs/Z-drugs and may imply that such use on a regular daily basis might lead to a faster development of drug dependence and a higher occurrence of adverse drug events.

All multiple regression models showed very similar results for association between country of residence and regular use of BZDs/Z-drugs, BZDs only or Z-drugs only. Patients residing in countries with the highest prevalence of particular medication group had higher probability to be prescribed a drug from this drug class. Our results suggest that even after adjustment for sociodemographic, clinical and functional status characteristics of NH residents, the country of residence remained strongly associated with the use of drugs from these medication groups. This may implicate that more country related non-clinical factors substantially influence prescription of BZDs and Z-drugs. Therefore, further specific studies in this field should be conducted.

Comparing our results with other studies, it is evident that not only clinical, but also ethnicity and historical aspects, as well as social, behavioural and cultural factors play an important role in the extensive use of BZDs/Z-drugs. In agreement with the findings of our study, a recent analysis showed 59 and 72% of long-term users of BZDs/Z-drugs among Israeli adults aged 65+ and 85+ years, respectively [40]. According to some studies, Israeli Arabs were significantly less likely to be prescribed BZDs compared to Israeli Jews [41] attributing these disparities to the post-traumatic stress experienced during the Holocaust [41]. On the other hand, the lower prescription of BZDs in Israeli Arabs might be associated with the stigmatising character of mental illnesses and a stronger reliability on informal support in this group [41]. Unfortunately, data about ethnicity and race were not collected in our study and we were not able to analyse the influence of these factors on the regular use of BZDs/Z-drugs in our study sample.

The differences in BZD/Z-drug prescribing patterns across countries may be partially explained also by the availability of particular BZDs/Z-drugs on the pharmaceutical market and by specific prescribing habits. For example, the most frequently prescribed BZD in Israel was brotizolam (59.8% of all BZDs/Z-drugs prescribed in Israeli NHs, and 99.4% of the overall brotizolam users in our sample). This correlates with the findings by Steinman et al., 2017 [40], which reported 46% of community dwelling older BZD users being prescribed brotizolam as the most common BZD in Israel. In contrary, despite availability on market, brotizolam was used just by 0.6% of Italian residents in our sample. Temazepam represented the most frequently prescribed BZD in the Netherlands, however, in spite of its availability on the pharmaceutical market, our study did not confirm to be prescribed in France. Also, other studies have found temazepam to be prescribed rarely (0.03%) in France [42]. Our findings, therefore, confirm importance of also country-specific prescribing patterns and habits for BZDs and Z-drugs.

There have been a number of warnings and campaigns against the inappropriate and excessive use of BZDs and Z-drugs. in participating countries. In France, detailed recommendations on how to help patients to withdraw from BZDs have been published by the Haute Autorité de Santé [43]. However, the overall consumption of BZDs/Z-drugs still remains high and sales of BZDs decreased by only 6.0% while sales of Z-drugs increased by 4.7% between 2005 and 2011 [10]. Pay-for-performance intervention in BZD prescription, recently introduced in France, has failed to help reduce the customarily extensive use of BZDs in this country, in the general population as well as in older adults [44].

In England, on the other hand, following recommendations to restrict BZD and Z-drug use in 2004 made by the Department of Health [45], led to decreased BZD consumption by 31.7% between 2005 and 2010 [10]. However, despite the recommendations given by the National Institute for Health and Care Excellence on the use of Z-drugs in 2004 [46], there was an increase of 7.3% in the prevalence of Z-drug use, mainly zopiclone [10]. This corresponds with the findings of our study, where zopiclone users represented 61.4% of the Z-drug users in England.

Since the 1980s, the German Federal Institute for Drugs and Medical devices has restricted the use of BZDs in Germany to a maximum period of 2–4 weeks, with the possibility of extension on an individual basis in cases of valid reasons [47]. The overall prevalence of BZD use in Germany decreased from 8.9 to 7.4% between 2006 and 2010 in the population aged 60–74 years, and from 13.3 to 10.4% in older adults aged ≥75 years [48]. In contrast, the prevalence of Z-drug use was relatively stable between 2006 and 2010, with a change from 2.1 to 2.0% in cohorts aged 60–74 years, and from 3.2 to 3.0% in older adults aged ≥75 years [48]. Given the restrictive character of regulatory interventions, it can be hypothesised that such regulations have a stronger impact on prescribing patterns compared to health campaigns or guideline recommendations. Thus, also in our study sample, more strict regulatory interventions might have a more positive influence on decreasing the regular use of BZDs/Z-drugs in Germany and England compared to other countries without strict regulations concerning BZD/Z-drug prescription.

The significant differences in the prevalence of BZD/Z-drug use in our samples of NH residents from different European countries and Israel document the strong role played by prescribing habits, social, cultural and also behavioural factors. In our study, the appropriateness of BZD/Z-drug use was not judged by thoroughly evaluating the quality of drug regimens under consideration of all relevant clinical and non-clinical factors. Therefore, we were not able to conclude on the real appropriateness of the use of these medications. However, serious discrepancies in findings on both the international and national levels warrant more attention and require the introduction of adequate measures to reduce the unnecessary use of BZDs/Z-drugs in European and Israeli NHs. Our multiple regression models confirmed that cross-national differences played a crucial role.

Given the risk/benefit ratio of BZDs/Z-drugs in the older population, other treatment options with a better safety profile should be considered in different indications in this population. Selective serotonin reuptake inhibitors, serotonin and norepinephrine reuptake inhibitors, and buspirone have been suggested as a pharmacologic treatment option for anxiety disorders and/or insomnia in these patients [9]. Also, non-pharmacological approaches, such as cognitive behavioural therapy, relaxation therapy, sleep hygiene, psycho-social support, etc. should not be underestimated or omitted in the treatment of insomnia in older adults. The research shows these interventions are highly beneficial in particular groups of patients [9, 11].

Europe currently represents a mix of highly heterogenic healthcare systems and national regulations concerning drugs. We are not aware of any current pan-European regulatory or clinical initiative that would help to assure the appropriate and necessary use of BZDs/Z-drugs in older adults in European NHs. A currently ongoing research project The EUROAGEISM project (ESR7/FIP7 subproject) [49] is targeted mainly on description of prescribing patterns in the use of PIMs in older adults 65+ in 6 Central and Eastern European countries and 2 developing countries. As mentioned above, among the most commonly prescribed PIMs at the EU level are often currently described BZDs/Z-drugs, thus this project will analyse also patterns of use of these medications. In collaboration with several policy partners (WHO, UNECE, Age Platform Europe and other policy partners), the project is also targeted to promoting of several policy strategies in order to reduce still excessive prescribing of PIMs (and also BZDs/Z-drugs) in different European countries.

Another, newly financed EU research project, iCARE4OLD (www.cordis.europa.eu/project/id/965341), will focus on analysing the comprehensive characteristics, outcomes, health trajectories and needs of older persons in European home care and NHs over the next 5 years (2021–2025). One of the WPs of this project deals with inappropriate prescribing including PIMs and BZD/Z-drug use, and will focus on describing the negative impact of pharmacological factors on older patients’ health trajectories in long-term care and determinants of their well-being, health and quality of life using IT artificial intelligence models and technologies.

### Study strengths and limitations

This study has several strengths and limitations. It comprised a large international sample of older people who had been residing for a long-term in NH facilities in multiple regionally different sites in the participating countries. Moreover, a substantial number of older long-term NH residents (450–500 residents per country) were prospectively assessed using a comprehensive geriatric assessment method. Using the same comprehensive methodology at all study sites enabled us to make meaningful comparisons of the current situation in the use of BZDs/Z-drugs in NH residents in the participating countries. Our study utilised a standardised, validated interRAI LTCF assessment tool consisting of different validated prospective geriatric scales of older adults’ clinical symptoms, syndromes, other clinical characteristics, and care needs. Thus, the diagnoses tested in our multiple regression models were not simply recorded from medical charts (which is usually an unprecise method) but were clarified by prospective geriatric clinical assessments conducted by trained clinical staff. The interRAI LTCF geriatric assessment has been previously validated in many European countries and is nowadays promoted and often applied in multidisciplinary clinical and social research. It is also already being used in clinical practice in some EU countries (e.g., Belgium and the Netherlands). Our study assessed all NH residents during the same time period, which enabled a unique, cross-national comparison of BZD/Z-drug prescribing patterns.

The main limitation of our study is that our sample was not selected to be representative of all NHs in each country [23], thus the residents’ characteristics cannot necessarily be generalised to all NH residents in the participating countries. There is a lack of other multicentric European studies in a NH setting that would enable a comparison of the major characteristics of older NH residents in order to identify similarities or discrepancies with our study. Populations of older adults in European NHs should be more thoroughly studied in future research to better describe their national characteristics, as they are one of the most frail and vulnerable populations of older adults in Europe. The SHELTER study was one of the first international studies focusing on making a description of comprehensive geriatric characteristics of vulnerable NH residents in selected samples in EU countries and Israel.

Another limitation of our study is that the cross-sectional design did not allow us to compare our results in a time-dependent manner, or to identify causal time relationships between BZD/Z-drug use and factors included in the multiple regression models. Therefore, we are not able to fully evaluate if the tested clinical factors are rather predictors or consequences of BZD/Z-drug use. Due to the cross-sectional design of our data and the aims of our study, we did not focus on evaluating longitudinal trends in BZD/Z-drug use. As all PRN BZDs/Z-drugs were excluded from our analytical models, this study somewhat underestimates the overall prevalence of BZD/Z-drug use. However, it is the regularity and long-term exposure to these medications that are commonly associated with drug dependence and other adverse drug events in this vulnerable older population. Therefore, the intention of our analyses focused specifically on the phenomenon of regular drug use.

As the main focus of this study was to describe the use of BZDs and Z-drugs, other drugs prescribed for similar indications (e.g., hydroxyzine, buspirone, mirtazapine, trazodone, risperidone, and others) were not analysed. Due to the character of the InterRAI LTCF assessment tool, it was also not possible to assess the utilisation of non-pharmacological treatment therapies or to investigate the appropriateness of BZD/Z-drug use in our study sample.

This study did not control for all clinical comorbidities. However, in the analyses of our multiple regression model, we included main comorbidities and factors associated with BZD/Z-drug use, e.g., anxiety and insomnia disorders, cognitive, functional and mood disorders, as well as pain and communication problems, which are currently also recognised as risk factors of BZD/Z-drug prescription.

## Conclusions

This study on older NH residents showed significant differences in the prevalence of BZD/Z-drug use across study samples selected from several European countries and Israel. It documented specific prescribing patterns analysed in country samples, as well as important associations between the extent of BZD/Z-drug use with country of residence. The results also suggested that non-clinical factors (prescribing habits, social, cultural, economic and behavioural factors, etc.) and regulatory and policy interventions significantly contribute to the current use of BZDs/Z-drugs and that there are significant differences in the prescribing patterns of these medications across Europe.

Given the current sparsity of data regarding BZD/Z-drug use in the specific population of older adults residing in NHs, it is of particular importance to further investigate those factors associated with and contributing to the unnecessary use of BZDs/Z-drugs on both the national and European levels. It is also very important to acknowledge the urgency of highly individually tailored treatments, supported by non-pharmacological interventions and regular professional medication checks. Such a systemic approach would help to individualise drug schemes and avoid unnecessary long-term use of BZDs/Z-drugs and other risks of therapy associated with these medications (e.g., non-geriatric dosing, drug-disease interactions, drug dependence, etc.).

## Supplementary Information

**Additional file 1: Table 1.** List of ATC codes and drug names included into the analyses within the dataset.

**Additional file 2: Table 2.** Proportion of the regular use of BZDs/Z-drugs and their combinations in the studied sample [50].

**Additional file 3: Table 3.** Factors influencing regular use of BZDs/Z-drugs – results from univariate logistic regression model.

## Data Availability

The datasets used and/or analysed during the current study are available from the corresponding author upon reasonable request.

## Abbreviations

ADLH: Activities of Daily Living Hierarchy scale
ATC: Anatomical Therapeutical and Chemical code
BZD(s): Benzodiazepines(s)
CAP: Clinical Assessment Protocol
CI: Confidence interval
CPS: Cognitive Performance Scale
DRS: Depression Rating Scale
EU: The European Union
IT: Information Technology
LTCF: Long-Term Care Facilities
NH(s): Nursing Home(s)
OR: Odds Ratio
PIM(s): Potentially Inappropriate Medication(s)
PRN: Per re nata – used on “as needed” basis
SHELTER: Services and Health in the Elderly in Long TERm care
WHO: World Health Organization
