# Supplementary material for: The prevalence and prescribing patterns of benzodiazepines and Z-drugs in older nursing home residents in different European countries and Israel: retrospective results from the EU SHELTER study
Source: BMC Geriatr. 2021 Apr 26;21:277. doi: 10.1186/s12877-021-02213-x (PMC8077828; doi:10.1186/s12877-021-02213-x)
Supplement: Supplementary file 1 — Additional file 1: Table 1. List of ATC codes and drug names included into the analyses within the dataset. [file 12877_2021_2213_MOESM1_ESM.docx]

# Title: The prevalence and prescribing patterns of benzodiazepines and Z-drugs in older nursing home residents in different European countries and Israel: retrospective results from the EU SHELTER study

**Running head:** Benzodiazepines/Z-drugs in European nursing homes

**Authors:**

Anna Lukačišinová^1^; Daniela Fialová^1,2^; Nancye May Peel^3^; Ruth Eleanor Hubbard^3^; Jovana Brkic^1^; Graziano Onder^4^; Eva Topinková^2^; Jacob Gindin^5^; Tamar Shochat^6^; Leonard Gray^3^; Roberto Bernabei^7^

**Affiliations:**

^1^ Department of Social and Clinical Pharmacy, Faculty of Pharmacy in Hradec Králové, Charles University, Hradec Králové, Czech Republic

^2^ Department of Geriatrics, 1^st^ Faculty of Medicine, Charles University, Prague, Czech Republic

^3^ Centre for Health Services Research, The University of Queensland, Brisbane, Australia

^4^ Department of Cardiovascular, Endocrine-Metabolic Diseases and Aging, Istituto Superiore di Sanità, Rome, Italy

^5^ The Center for Standards in Health and Disability, The University of Haifa, Haifa, Israel

^6^ The Cheryl Spencer Department of Nursing, The University of Haifa, Haifa, Israel

^7^ Centro Medicina dell’Invecchiamento, Dipartimento di Scienze Gerontologiche, Geriatriche e Fisiatriche, Universita Cattolica Sacro Cuore, Rome, Italy

**Corresponding Author:**

Anna Lukačišinová, PharmD., Ph.D.

Department of Social and Clinical Pharmacy

Faculty of Pharmacy in Hradec Králové

Akademika Heyrovského 1203

500 05 Hradec Králové

Czech Republic

E-mail: lukacisinova.anna@gmail.com

Telephone Number: +420 774 938 108

ORCID: 0000-0001-6461-5977

**Additional Table 1** List of ATC codes and drug names included into the analyses within the dataset.

| **ATC code** | **Drug name** |
| --- | --- |
| *Benzodiazepine anxiolytics* | |
| N05BA01 | Diazepam |
| N05BA02 | Chlordiazepoxid |
| N05BA03 | Medazepam |
| N05BA04 | Oxazepam |
| N05BA05 | Potassium Clorazepate |
| N05BA06 | Lorazepam |
| N05BA07 | Adinazolam |
| N05BA08 | Bromazepam |
| N05BA09 | Clobazam |
| N05BA10 | Ketazolam |
| N05BA11 | Prazepam |
| N05BA12 | Alprazolam |
| N05BA13 | Halazepam |
| N05BA14 | Pinazepam |
| N05BA15 | Camazepam |
| N05BA16 | Nordazepam |
| N05BA17 | Fludiazepam |
| N05BA18 | Ethyl Loflazepate |
| N05BA19 | Etizolam |
| N05BA21 | Clotiazepam |
| N05BA22 | Cloxazolam |
| N05BA23 | Tofisopam |
| N05BA56 | Lorazepam, combinations |
| *Benzodiazepine hypnotics* | |
| N05CD01 | Flurazepam |
| N05CD02 | Nitrazepam |
| N05CD03 | Flunitrazepam |
| N05CD04 | Estazolam |
| N05CD05 | Triazolam |
| N05CD06 | Lormetazepam |
| N05CD07 | Temazepam |
| N05CD08 | Midazolam |
| N05CD09 | Brotizolam |
| N05CD10 | Quazepam |
| N05CD11 | Loprazolam |
| N05CD12 | Doxefazepam |
| N05CD13 | Cinolazepam |
| *Z-drugs* | |
| N05CF01 | Zopiclone |
| N05CF02 | Zolpidem |
| N05CF03 | Zaleplon |
| N05CF04 | Eszopiclone |
