# Supplementary material for: The prevalence and prescribing patterns of benzodiazepines and Z-drugs in older nursing home residents in different European countries and Israel: retrospective results from the EU SHELTER study
Source: BMC Geriatr. 2021 Apr 26;21:277. doi: 10.1186/s12877-021-02213-x (PMC8077828; doi:10.1186/s12877-021-02213-x)
Supplement: Supplementary file 2 — Additional file 2: Table 2. Proportion of the regular use of BZDs/Z-drugs and their combinations in the studied sample [50]. [file 12877_2021_2213_MOESM2_ESM.docx]

# Title: The prevalence and prescribing patterns of benzodiazepines and Z-drugs in older nursing home residents in different European countries and Israel: retrospective results from the EU SHELTER study

**Running head:** Benzodiazepines/Z-drugs in European nursing homes

**Authors:**

Anna Lukačišinová^1^; Daniela Fialová^1,2^; Nancye May Peel^3^; Ruth Eleanor Hubbard^3^; Jovana Brkic^1^; Graziano Onder^4^; Eva Topinková^2^; Jacob Gindin^5^; Tamar Shochat^6^; Leonard Gray^3^; Roberto Bernabei^7^

**Affiliations:**

^1^ Department of Social and Clinical Pharmacy, Faculty of Pharmacy in Hradec Králové, Charles University, Hradec Králové, Czech Republic

^2^ Department of Geriatrics, 1^st^ Faculty of Medicine, Charles University, Prague, Czech Republic

^3^ Centre for Health Services Research, The University of Queensland, Brisbane, Australia

^4^ Department of Cardiovascular, Endocrine-Metabolic Diseases and Aging, Istituto Superiore di Sanità, Rome, Italy

^5^ The Center for Standards in Health and Disability, The University of Haifa, Israel

^6^ The Cheryl Spencer Department of Nursing, The University of Haifa, Israel

^7^ Centro Medicina dell’Invecchiamento, Dipartimento di Scienze Gerontologiche, Geriatriche e Fisiatriche, Universita Cattolica Sacro Cuore, Rome, Italy

**Corresponding Author:**

Anna Lukačišinová, PharmD., Ph.D.

Department of Social and Clinical Pharmacy

Faculty of Pharmacy in Hradec Králové

Akademika Heyrovského 1203

500 05 Hradec Králové

Czech Republic

E-mail: lukacisinova.anna@gmail.com

Telephone Number: +420 774 938 108

ORCID: 0000-0001-6461-5977

**Additional Table 2** Proportion of the regular use of BZDs/Z-drugs and their combinations in the studied sample

| **Drug/drug group** | **Number of BZD/Z-drug users** | **Proportion of BZD/Z-drug users (%) from the total sample** | **Proportion of BZD/Z-drug users (%) from *all* BZD/Z-drug users** |
| --- | --- | --- | --- |
| **All BZDs and Z-drugs** | 1,440 | 35.8 | 100.0 |
| **All regular BZDs and Z-drugs ^a^** | 1,113 | 27.7 | 77.3 |
| BZDs | 897 | 22.3 | 62.3 |
| Z-drugs | 328 | 8.1 | 22.7 |
| 2 different BZDs combination | 51 | 1.3 | 3.5 |
| BZDs and Z-drugs combination | 61 | 1.5 | 4.2 |
| 2 BZDs and 1 Z-drug combination | 1 | 0.02 | 0.1 |
| **All regularly used BZDs and Z-drugs divided by half-life ^b, c^** | | | |
| ***Short and intermediate half-life BZDs*** *(≤ 25 h including active metabolite)* | | | |
| Lorazepam^*^ | 190 | 4.7 | 17.1 |
| Oxazepam^*^ | 182 | 4.5 | 16.3 |
| Alprazolam^*^ | 115 | 2.9 | 10.3 |
| Temazepam | 106 | 2.6 | 9.5 |
| Bromazepam^*^ | 39 | 0.9 | 3.5 |
| Midazolam | 23 | 0.6 | 2.1 |
| Lormetazepam | 9 | 0.2 | 0.8 |
| Triazolam | 9 | 0.2 | 0.8 |
| Tofisopam | 2 | 0.05 | 0.2 |
| ***Long half-life BZDs*** *(> 25 h including active metabolite)* | | | |
| Brotizolam | 154 | 3.8 | 13.8 |
| Diazepam^*^ | 34 | 0.8 | 3.1 |
| Nitrazepam | 7 | 0.2 | 0.6 |
| Clobazam | 4 | 0.1 | 0.4 |
| Flunitrazepam | 3 | 0.1 | 0.3 |
| Flurazepam | 2 | 0.05 | 0.2 |
| Potassium Clorazepate | 2 | 0.05 | 0.2 |
| Prazepam | 1 | 0.02 | 0.1 |
| ***Z-drugs*** | | | |
| Zopiclone | 198 | 4.9 | 17.8 |
| Zolpidem | 130 | 3.2 | 11.7 |
| ***Additional information on PRN use of BZDs and Z-drugs in the studied sample*** | | | |
| ***All PRN BZDs and Z-drugs*** | *327* | *8.1* | *22.7* |
| *BZDs* | *293* | *7.3* | *20.3* |
| *Z-drugs* | *71* | *1.8* | *4.9* |
| *PRN BZD/Z-drug and regular BZD/Z-drug combination* | *74* | *1.8* | *6.6* |
| *2 different BZDs combination* | *17* | *0.4* | *1.2* |
| *BZDs and Z-drugs combination* | *20* | *0.5* | *1.3* |

PRN – *per re nata* – medication prescribed on “as needed” basis

^a^ Total number of regular BZD/Z-drugs users is counted as sum of patients using BZD/Z-drugs regularly, regardless their combinations across drugs or duplicities within or one drug. In this variable every patient is counted just once as users of BZD/Z drugs. Out of 1113 regular users, 113 patients used combinations of 2 different BZDs, 1 BZD and 1 Z-drug or 2 BZDs and 1 Z-drug. In total, 1247 BZDs and Z-drugs were prescribed in the sample for regular use (including combinations of different drugs and combinations of same drug). For duplicities of one drug in prescription of one patient see footnote for a particular drug in the table (*).

^b^ Biological half-life of BZDs was derived from Ashton, 2002 [50]

^c^ In the section of All regularly used BZDs and Z-drugs divided by half-life, the prevalence of BZDs and Z-drugs given in the last column represents proportion of BZD/Z-drug users (%) from all regular BZD/Z-drug users.

^*^ In total, 20 patients used 2 same BZDs in two different drug products/brand names at the same time: lorazepam – 7 patients; oxazepam – 4 patients; alprazolam – 5 patients; bromazepam – 1 patient; diazepam – 3 patients. One patient used 3 oxazepams at the same time.
