# Supplementary material for: The prevalence and prescribing patterns of benzodiazepines and Z-drugs in older nursing home residents in different European countries and Israel: retrospective results from the EU SHELTER study
Source: BMC Geriatr. 2021 Apr 26;21:277. doi: 10.1186/s12877-021-02213-x (PMC8077828; doi:10.1186/s12877-021-02213-x)
Supplement: Supplementary file 3 — Additional file 3: Table 3. Factors influencing regular use of BZDs/Z-drugs – results from univariate logistic regression model. [file 12877_2021_2213_MOESM3_ESM.docx]

# Title: The prevalence and prescribing patterns of benzodiazepines and Z-drugs in older nursing home residents in different European countries and Israel: retrospective results from the EU SHELTER study

**Running head:** Benzodiazepines/Z-drugs in European nursing homes

**Authors:**

Anna Lukačišinová^1^; Daniela Fialová^1,2^; Nancye May Peel^3^; Ruth Eleanor Hubbard^3^; Jovana Brkic^1^; Graziano Onder^4^; Eva Topinková^2^; Jacob Gindin^5^; Tamar Shochat^6^; Leonard Gray^3^; Roberto Bernabei^7^

**Affiliations:**

^1^ Department of Social and Clinical Pharmacy, Faculty of Pharmacy in Hradec Králové, Charles University, Hradec Králové, Czech Republic

^2^ Department of Geriatrics, 1^st^ Faculty of Medicine, Charles University, Prague, Czech Republic

^3^ Centre for Health Services Research, The University of Queensland, Brisbane, Australia

^4^ Department of Cardiovascular, Endocrine-Metabolic Diseases and Aging, Istituto Superiore di Sanità, Rome, Italy

^5^ The Center for Standards in Health and Disability, The University of Haifa, Israel

^6^ The Cheryl Spencer Department of Nursing, The University of Haifa, Israel

^7^ Centro Medicina dell’Invecchiamento, Dipartimento di Scienze Gerontologiche, Geriatriche e Fisiatriche, Universita Cattolica Sacro Cuore, Rome, Italy

**Corresponding Author:**

Anna Lukačišinová, PharmD., Ph.D.

Department of Social and Clinical Pharmacy

Faculty of Pharmacy in Hradec Králové

Akademika Heyrovského 1203

500 05 Hradec Králové

Czech Republic

E-mail: lukacisinova.anna@gmail.com

Telephone Number: +420 774 938 108

ORCID: 0000-0001-6461-5977

**Additional Table 3** Factors influencing regular use of BZDs/Z-drugs – results from univariate logistic regression model

| **Factors influencing prescription** | **Unadjusted OR** | **95% CI** | **p value** |
| --- | --- | --- | --- |
| **Age** | | | |
|  | 0.994 | 0.987 - 1.001 | 0.10 |
| **Gender** | | | |
| Male – reference | - | - | - |
| Female | 1.047 | 0.895 - 1.225 | 0.57 |
| **Country ordered by increasing prevalence of BZD/Z-drug use** | | | |
| Germany – reference | 1.000 | - | - |
| England | 1.237 | 0.881 - 1.737 | 0.22 |
| Czech Republic | **1.491** | **1.070 - 2.077** | **0.02** |
| Finland | **1.572** | **1.128 - 2.191** | **0.008** |
| Italy | **2.226** | **1.645 - 3.122** | **<0.001** |
| The Netherlands | **2.489** | **1.814 - 3.416** | **<0.001** |
| France | **4.630** | **3.407 - 6.292** | **<0.001** |
| Israel | **4.653** | **3.451 - 6.273** | **<0.001** |
| **CPS^a^** | **0.933** | **0.900 - 0.968** | **<0.001** |
| **ADLH^b^** | **0.983** | **0.976 - 0.990** | **<0.001** |
| **Pain scale^c^** | **1.228** | **1.130 - 1.336** | **<0.001** |
| **CAP Delirium^d^** | 1.052 | 0.965 - 1.146 | 0.25 |
| **Depression scale^e^** | **1.088** | **1.061 - 1.115** | **<0.001** |
| **Communication scale^f^** | **0.994** | **0.921 - 0.968** | **<0.001** |
| **Anxiety^g^** | | | |
| Not present – reference | 1.000 | - | - |
| Diagnosis present | 1.627 | 0.909 - 2.913 | 0.10 |
| **Diagnosis present, treated** | **2.956** | **2.308 - 3.785** | **<0.001** |
| Diagnosis present, monitored | 1.367 | 0.944 - 1.980 | 0.10 |
| **Difficulty falling asleep** | | | |
| Not present – reference | 1.000 | - | - |
| **Present, not exhibited** | **2.519** | **2.026 - 3.134** | **<0.001** |
| **Exhibited 1 of 3 days** | **2.106** | **1.483 - 2.990** | **<0.001** |
| **Exhibited 2 of 3 days** | **1.830** | **1.208 - 2.770** | **0.004** |
| **Exhibited daily of 3 days** | **3.244** | **2.549 - 4.129** | **<0.001** |

Results in **bolt** indicate statistically significant results

^a^ CPS– Cognitive Performance Scale [26] was used to access cognitive status. It includes five items: cognitive skills for daily decision making, short-term memory problems, procedural memory problems, making self-understood, and eating ability. Scores of CPS items range from 0 (intact) to 6 (very severe cognitive impairment), and any score ≥2 indicates clinically significant cognitive impairment (from mild to very severe stages).

^b^ ADLH scale –Activities of Daily Living Hierarchy scale [25] comprises 7 items: personal hygiene, dressing upper body, dressing lower body, locomotion, toilet use, bed mobility, eating. Each item is scored from 1 = requires supervision to 4 = total dependence. The scale ranges from 0 to 28, with higher scores reflecting greater level of dependency and difficulties in performing activities.

^c^ Pain scale [28] - summarizes the reported presence and intensity of pain. It comprises two items: pain symptoms-frequency and pain symptoms-intensity of highest level of pain present. The scores range from 0 =no pain to 4 =daily excruciating pain

^d^ CAP Delirium [30] - this scale comprises 4 items: easily distracted, disorganized speech, mental function varies over day, change in decision making. The scale ranges from 0 to 4, with higher values indication increase likelihood of delirium.

^e^ Depression scale [27] - is based on the self-reported mood items and indicates the presence of depressed mood and anxiety. It consists of 3 self-reported mood items, while each question can be scored from 0 to 2 with the maximum overall score of 6. The score of this scale range from 0 = no symptoms of depression to 6 = all symptoms present in last 3 days/24 hours: high likelihood of depression.

^f^ Communication scale [29] – consists of two items: making self-understood (expression) and ability to understand others (comprehension), while not taking directly into consideration hearing and visual impairment. It is primarily focused on dysphasia and similar syndromes. The scores range from 0 = intact to 8 = very severe impairment.

^g^ “Diagnosis present” – recorded when diagnosis confirmed as diagnosed clinical condition in medical charts; “Diagnosis present, treated” – resident’s diagnosis is being treated by active treatment (incl. drug therapy, therapeutic rehabilitation services, other medical or skilled nursing interventions); “Diagnosis present, monitored” – resident’s diagnosis is being only monitored (e.g., by laboratory tests, vital signs, etc.) but no active treatment is provided.
